# Causality in Psychiatry: A Hybrid Symptom Network Construct Model

**DOI:** 10.3389/fpsyt.2015.00164

**Published:** 2015-11-20

**Authors:** Gerald Young

**Affiliations:** ^1^York University, Toronto, ON, Canada

**Keywords:** causality, symptom, mental disorder, construct, network

## Abstract

Causality or etiology in psychiatry is marked by standard biomedical, reductionistic models (symptoms reflect the construct involved) that inform approaches to nosology, or classification, such as in the DSM-5 [Diagnostic and Statistical Manual of Mental Disorders, Fifth Edition; (1)]. However, network approaches to symptom interaction [i.e., symptoms are formative of the construct; e.g., (2), for posttraumatic stress disorder (PTSD)] are being developed that speak to bottom-up processes in mental disorder, in contrast to the typical top-down psychological construct approach. The present article presents a hybrid top-down, bottom-up model of the relationship between symptoms and mental disorder, viewing symptom expression and their causal complex as a reciprocally dynamic system with multiple levels, from lower-order symptoms in interaction to higher-order constructs affecting them. The hybrid model hinges on good understanding of systems theory in which it is embedded, so that the article reviews in depth non-linear dynamical systems theory (NLDST). The article applies the concept of emergent circular causality (3) to symptom development, as well. Conclusions consider that symptoms vary over several dimensions, including: subjectivity; objectivity; conscious motivation effort; and unconscious influences, and the degree to which individual (e.g., meaning) and universal (e.g., causal) processes are involved. The opposition between science and skepticism is a complex one that the article addresses in final comments.

## Causality in Psychiatry: A Hybrid Symptom Network Construct Model

The article tackles fundamental issues in psychiatry while proposing novel solutions. In particular, it considers the relationship between symptoms and disorder by examining extant models and current research. It attempts to disambiguate some of the confusions related to understanding and researching the models, preparing the way for presentation of a genuinely hybrid one based on systems theory thinking. Moreover, it presents other novel concepts related to emergent causality, and the relationship of meaning and causality in symptoms (hermeneutic insight and causal explanation; Verstehen, Erklären, respectively). The article presents a complex view of the relationship between meaning and causality involving three dimensions.

## Introduction

### Opposing Models

The article reflects on two types of models, a latent variable model (or construct model), which is seen as a top-down approach to understanding the relationship between symptoms and disorders, and a symptom interaction model (or network model), which is seen as a bottom-up approach to understanding the relationship between symptoms and disorders. In latent variable modeling, an underlying construct (e.g., depression) is considered causal of the relationship of the items or behaviors (e.g., symptoms) that are subsumed by the variable. In an item or behavior interaction or networked model (e.g., symptoms), relatively few direct relations are considered causal of the item/behavior/symptom relationships, which are deemed to lie among the latter themselves.

In the first model of the two involved, which is the traditional approach, symptoms (items) reflect a common underlying psychological construct and, therefore, this type of model is considered “reflective” (4). In this construct model, the cause of the mental symptom/disorder derives from the central construct, whether a disorder or a cluster, downward to the symptoms. In the symptom-interactive model, symptoms (items) mutually affect each other, and can be represented by a composite variable, but the direction of the causality is from the symptom interactions to the composite. The model is referred to as “formative” (4).

In this network model, which is the second of the two involved, causality springs from the symptoms (or clusters) interacting among themselves, a process that acts to change the symptoms/clusters (or initiate them). The composite variable is involved only as representation.

Before describing the hybrid model in depth, some of the challenges in doing so are described. This leads to presentation of a literature review preparatory to it.

### Systems

The article will consider the following crucial questions. First, what do we miss when we represent disorders solely with top-down models such as the construct model? What do we miss when we represent disorders solely with bottom-up models such as a network model? In order to answer these questions, the hybrid model that has been created is framed in Non-Linear Dynamical Systems Theory (NLDST), which can be viewed both as a model that is an umbrella one or superordinate one to the construct and network ones. Therefore, the article presents a novel hybrid model, which combines these two types of models (top-down, bottom-up) into a framework that both respects them yet adds to them without detracting from them.

In this work, researchers might obtain a covariance matrix related to the multiple symptoms in a study (referring to the covariance among scores of participants with respect to the symptoms that were measured). Once the matrix is established, the covariance obtained could be explained from either a common construct perspective or from that of symptom network interactions (i.e., common cause vs. direct causal relations). In this regard, the researcher evaluates either (a) the shared variance of all measured variables of a putative construct, e.g., estimating factor loadings, and the causal pattern is from the construct to the variables; or (b) the parameters for the direct relations between symptoms. Furthermore, in one type of hybrid approach, the variance that is not explained by the common construct might be explained residually through direct relations between networked symptoms.

That being said, the present hybrid model is not built on statistical synergies but conceptual ones. It presents a theoretically plausible causal model and the statistical task, then becomes to fit extant statistical approaches to the model or expand them for this purpose. The conceptual hybrid model is built on NLDST, and the multilevel hierarchical structure that it includes allows for upper levels of the system to work with lower levels in establishing the system whole. That is, if we equate psychological constructs with emergent higher-order system levels that might derive from lower-order levels and their bottom-up interactions, such as in networks, then the stage is set for having higher-order levels reciprocally influence in turn in a top-down fashion the networks involved. For example, depression might not only be constructed by its symptoms but also it might exist as a subjective mental content or disorder and influence the configuration of its symptoms (in context, and for the individual in her/his uniqueness).

If one excludes psychological constructs from consideration as a higher-order level in a systems model, the hybrid model as presented will be dismissed. However, if one allows for its inclusion in a systems framework, as described, the framework can readily be conceived as one that has emergent higher-order levels (or constructs, e.g., mental content and disorder) that can interact top-down and reciprocally with symptom networks in their bottom-up influence on the system.

### Clusters

Another complication in developing a hybrid model involving construct and network approaches to symptom–disorder relationships involves clusters, which stand intermediate between symptoms and disorders. In network modeling, subsets (clusters) of items, behaviors, or symptoms (variables) might be found, but they are not considered as independent sources of causation relative to the direct relationships among the variables. Rather, variables within any one cluster might causally influence each other in their network. Inter-variable correlations will result, but they would not reflect the causal influence of a common underpinning construct. The article will deal with this issue as it proceeds in creating a genuine hybrid model.

On the one hand, the DSM-5 [Diagnostic and Statistical Manual of Mental Disorders, Fifth Edition; (1)] includes many disorders that, through its polythetic approach to symptom identification, involve symptom clusters. However, the research on how many clusters are needed in the DSM’s disorders stands as an ongoing enterprise. For example, in posttraumatic stress disorder (PTSD), the empirical findings on how to group its symptoms keeps finding an increasing amount of clusters. The number of factors or dimensions involved in research on PTSD has moved it from the DSM-5’s four-dimensional model to ones with even seven and eight dimensions (see below). On the other hand, in systems modeling, there is no reason why intermediate levels cannot constitute both top-down causal levels working on lower ones and levels that can be influenced by those lower ones, while having levels superordinate to them influence them, while they form networks among themselves that can influence their superordinate levels. Therefore, systems modeling can accommodate the concept of clusters in symptoms.

The final prefatory note about the present hybrid model of symptom–mental disorder relations (with its mutual bottom-up and top-down influences) is that its hybrid nature is not synonymous with an attempt to explain everything related to the question to the point that its inclusivity can really explain nothing. In this regard, there are many multifactorial models in causal explanation that are acceptable; there are many systems models in this regard; and there are many advancing conceptual and statistical notions that are hybrid and explanatory without being obtuse and untestable [e.g., Ref. (5, 6)].

## Issues

In the following, the first substantive section of the article considers relevant concepts and terms. This lays the foundation for the literature review, model building, and applications.

### Concepts

Psychiatry has been criticized at multiple levels, including its difficulties with its diagnostic manuals and their assumptions. It embraces mostly the medical model of disorder and diagnosis, the biocentric model of the causality of disorder, or etiology, and the psychopharmacological model of disorder treatment and management (6–9). Even the basic concept of what constitutes a mental disorder has been disputed. In the following, I review aspects of these issues, preparing the way for presentation of my own work in the area. The section ends with an integrated view of what is mental disorder.

The RDoC project [Research Domain of Criteria; (10, 11)] contends that it offers a broad approach to causation in psychiatry, but its critics maintain that it is especially biomedical, neurocentric, and reductionistic [e.g., Ref. (6, 12, 13)]. Similarly, the DSM-5 is a psychiatric classificatory system that aims to include reliable and valid categories of mental disorder with clear causes (etiology), an aspiration that, if realized, would facilitate effective treatment; however, its critics maintain that it fails to achieve its objective [e.g., Ref. (6–9)]. Also, in terms of causal explanations, they maintain that it is still steeped in the biocentric model.

Multifactorial causal models in psychiatry have been formulated, such as the biopsychosocial model [e.g., Ref. (5, 6)]. Moreover, newer modeling efforts are specifying the mechanisms in the interactions among causal influences on behavior and its disturbance, such as work on networks [e.g., Ref. (2, 14)] and attractor dynamics [e.g., Ref. (15)].

To highlight in more depth the main argument of the article, bottom-up causality in psychiatry refers to the interaction and mutual influence of symptoms in mental illness, while top-down ones refer to the influence of underlying latent variables or constructs on symptom expression. A genuinely interactive bottom-up, top-down model would acknowledge both the reality of an underlying latent variable or construct in influencing symptomatology and also networked symptom connections as influencing the underlying construct.

This approach might be antithetical to those who hold either a network or construct view of system–disorder relations, but there are advantages to the model. Moreover, it fits the overarching model of systems theory. In this regard, the next section of the article explains in depth the concept of systems, which includes different levels, self-organization, emergence, and attractors.

### Non-Linear Dynamical Systems Theory

This section of the article reviews some critical concepts in NLDST. Detailed presentation of systems theory is beyond the scope of the present work; the reader should consult Thelen and Smith (16); Young (3), and also Bielczyk et al. (15).

NLDST is distinguished by its emphasis on self-organized emergence in system component interactions within and across levels. In particular, higher-order levels of systems might emerge through bottom-up interactive processes. For example, Vallacher et al. (17) referred to the emergence of “global properties” or “coherent higher-order” states through the adjustment to each other of the individual system elements involved in a bottom to top (bottom-up, instead of top-down) self-organizational process. Typically, self-organization does not reach the new system end-state instantaneously. Rather, there are many ongoing mutual system element adjustments that take place.

Through its concepts of emergence and self-organization, NLDST allows for explanation of how higher-order patterns in behavior, from the simplest limb movements to the most profound thoughts, are part of the species’ repertoire. New systems states that emerge in a system function to constrain behavior emanating from the system. New state system input transforms toward state characteristics even if they are discrepant with them. That is, systems maintain stability once formed, even if perturbed, until further mutual element and input interactions lead to critical state transition points.

System states might change over time, but when they consistently return to the same state after perturbation, the state involved is considered an attractor. Attractors reside in landscapes with basins; and the wider are the basins, the more likely a range of states in the system will converge on one attractor, which metaphorically could be considered to reside at the bottom of the basin involved. In this model, the “deeper” is the basin, the greater is the attractor’s resistance to perturbation.

When a system has two or more states, it is considered multistable. The attractors could involve negative or undesirable states, such as having in the same person antagonism in conjunction with antagonism avoidance. Or, the two members of a couple could be living antagonistic regimes [e.g., Ref. (18)]. Beyond attractors on which system dynamics converge, an attractive force could be like a “repellor,” or one that scrupulously avoids regions in its state space rather than returning to it. Metaphorically, instead of in a basin, a repellor resides on top of a hill in the system’s landscape.

Systems might have no attractors, and therefore be more susceptible to external influences. Or, systems might have one attractor, sustain a perturbation that is critical (19), and produce self (re)organization through the effect on set points in control parameters in the system.

Finally for Vallacher et al. (17), dynamic properties can be found at “different levels of psychological reality,” and dynamic transformations can take place at different time scales (seconds, years). Also, network concepts fall under the rubric of dynamic ones. That is, network nodes represent elements in systems. This notion is important for present purposes in that it justifies considering network models as part of larger ones in NLDST that includes higher-order levels that can be represented as constructs.

Samuelson et al. (20) emphasized the relevance of emergence in NLDST. Emergence takes place through systems components that interact and mutually influence each other in a soft-assembly process, or from the ground, rather than from pre-specified central, top-down (deterministic) explicit coding or organization. It takes place over multiple time scales; can happen on the moment; and is conditioned by context and the history of the organism, so that the outcome is unique and variable. Systems might also have subsystems, which are strongly coupled or integrated components that are only weakly coupled at best to other components. The authors give the example of seeking hidden objects in a first location even after viewing its hiding in a second one. Research shows that, in infants, the error involved (A-not-B) is a product of cognitive and motor components in interaction, with temporal and neural dynamics at work, too.

Hayes et al. (21) noted that dynamic systems concern pattern formation and change. The principles in dynamic systems science cut across biology, ecology, political science, and other disciplines, including physics and chemistry. Systems are adaptive when they maintain a dynamic tension between stability and variability. Although resilience to perturbation can be beneficial, it should not be overly rigid. For example, from a network perspective, in depression, negative emotions exhibit stronger temporal connections (22). Psychotherapy can help in shifting maladaptive connections to adaptive ones, as demonstrated in the research of Hayes and colleagues and Schiepek et al. (23).

NLDST is a mathematical model that is conducive to psychological theorizing. For example, attractors can be represented by mathematical formalisms, and state spaces or trajectories in a system can be represented by graphical representation of differential equations (24). In this regard, modeling could include approaches such as dynamic factor analysis and application of ergodic theory (25). Butner et al. (24) explained that, mathematically, Lyapunov exponents represent the strength of system topological features, for example, the rate a system changes toward or away from a particular state (the basin steepness). They can be calculated locally (e.g., at a set point) or globally (for the whole system).

Rabinovich et al. (26) described dynamic transformation as allowing cognition and mind to emerge from brain and computation. Cognition is not reflected in any one brain center or even in the entire brain, but is a product of interconnected cooperativity over many elements. The spatiotemporal patterns in brain dynamics that are highly coherent could be called modes, and they reflect the play of extinction and stabilizing inhibition. Brain center clusters that form in tasks represent dynamical modes and correspond to transient system states. Superordinate levels in systems can be conceived as hierarchically arranged chunking networks, e.g., from sentences to paragraphs to chapters in texts.

Wichers et al. (27) indicated how moment-to-moment affect dynamics can be viewed from NLDST. They referred to research showing that symptom networks in (severe) psychopathology are more strongly interconnected than those of people with less severe psychopathology [e.g., Ref. (14)]. The networks exhibit vicious circles because their nodes reinforce each other. In dynamical terms, a system could be “very” stable and therefore even “strong” perturbations might not create variability, let alone a small one allowing for “critical transition” to another state at a “tipping point.” However, in psychopathology, if there is high, mutually reinforcing connectivity with networks, such that vicious circles develop in the background without being noticed, the mood system could become fragile and vulnerable to transition, even when one node (affective state) is triggered for the reason that others are also activated in the network. A cascade effect results that continues to resonate in the network such that the “little” perturbation of the one node involved leads to a disproportionate mood change or critical transition (as in the well-known butterfly effect).

### Mental Disorder and Symptoms

Before continuing in the article with the literature review and detailed modeling, the concept of a symptom needs clarification. This is undertaken toward better understanding mental disorder, which is also discussed in this section.

#### Symptom

A symptom is defied as a physical or mental feature that is a departure from a typical state or feeling and that might be indicative of a disease, disturbance, disorder, unusual state, or condition (and which might be noticed by the patient). Symptoms might be subjectively experienced and phenomenologically reported or objectively obtained (signs, e.g., in laboratory tests). Symptoms include the contents of mental states that might recursively influence other symptoms, such as through the vicious circles that take place after catastrophic thinking and fears. Beliefs are powerful engines driving symptomatology, as are moods, affect, emotions, drives, desires, and motivation.

Much of the work in psychotherapy relates to these mental contents, the narratives people tell about themselves and to others, and the meanings ascribed to events, as well as one’s past experiences, and one’s place in the present and future, in addition to other people’s actions and reactions to the person and their relationships with the person. In this sense, the person is as much, if not more, a seat of the causality of symptomatology experienced as are biological (nature) and environmental (nurture) factors (as per the biopsychosocial/biopersonalsocial models mentioned previously). That being said, not all symptoms can be taken at face value or are even genuine. This is especially true because of the influence of unconscious processes on symptom expression, as well as even conscious ones, such as those related to feigning or malingering for monetary gain, as might happen with PTSD, the exemplar chosen in the article. This difference between patient presentation and actual symptomatology constitutes the quandary confronting clinicians, as well as the challenge that they and their patients must work through.

#### Mental Disorder

As for defining mental disorder, there is no one accepted definition. The approach of the DSM-5 involves a clinically significant disturbance reflecting dysfunction usually associated with distress or disability in activity. In contrast, for the DSM, normally neither an expected, culturally approved loss to a common stressor/loss nor individual-society conflicts, involving socially deviant behavior, are representative of mental disorder.

The DSM-5’s definition of mental disorder includes an error in reasoning (8). It indicates tautologically that a mental disorder is caused by a disturbance in mental functioning, which simply uses the same words on both sides of the definitional equation. The World Health Organization (28) definition of mental disorder does not help resolve the matter. It refers to a disorder as a combination of thoughts, perceptions, emotions, behavior, and relationships that are “abnormal.”

Closer inspection of the DSM-5 definition of mental disorder indicates that it is constituted by different levels. The DSM-5 definition has implicit in it several levels. They include mental function atop the hierarchy, then mental disorder as one branch. The collection of signs (objective) and symptoms (subjective) happen behaviorally, emotionally (in regulation) and cognitively, and together constitute a syndrome. Furthermore, the ensemble of signs and symptoms are associated with a “clinically” significant disturbance and usually a “significant distress, or disability.” Finally, at another level implicating causality, there are “dysfunctions” in psychological, biological, or developmental “processes.” Aside from these levels, often, mental disorder in the DSM-5 includes clusters of symptoms intermediate between the disorder and symptom list.

Both the DSM-5 definition of mental disorder and the WHO’s definition do not include directly environment, support or its lack, or context. A relational and systemic approach to mental disorder might better arrive at its acceptable and inclusive definition [e.g., Ref. (8)].

Young (6) developed a more elaborative definition of mental disorder. According to him, it involves “a behavioral syndrome (or pattern or network of symptoms) in context that is characterized as a clinically significant disturbance, distress, or dysfunction potentially evaluated as harmful to the individual, to others, or to both.” To establish clinical significance, well-informed (and trained) individuals should rely on reliable and relevant evidence. The mental disorder can be expressed in cognition, mood, relations, interactions, self-regulation, and other behavior and its organization. Biological, social, and personal (i.e., psychological), as well as developmental processes, might be factors. Social, occupational, or other important functional activities might be involved in impairment, and they might meet disability thresholds. The definition of mental disorder that I have provided is based on the DSM’s approach, but broadens it, for example, by mentioning symptom networks, which is important in the present context.

#### Diagnostic and Statistical Manual of Mental Disorders, Fifth Edition

According to Vanheule (9), a diagnostic category needs to be both reliable and valid. For reliability, he noted that the range of kappa results in reliability studies of DSM categories has shifted in the qualitative attribute given to the best results, the next best, and so on. In particular, Clarke et al. (29) used a shift in describing kappa results that seemingly allowed for acceptable reliability for quite a few DSM-5 categories in the DSM-5 field trials, when use of the kappa ranges used in research on prior versions of the DSM would have shown questionable reliability for those DSM-5 results had the prior adjectives in summarizing kappa results had been applied without change. That being said, I note that the best results were obtained for PTSD (along with a few others; PTSD reliability results for the DSM-5 were considered at least fair or very good, depending on the criteria).

As for validity, Vanheule (9) queried whether the DSM-5 accounts well for context and whether its categories apply well to individual cases. He concluded that, rather than symptoms being signs or indices, they should be conceived as personal constructions.

The next section of the article examines recent literature related to topics in mental disorder. They include work on network models and the construct approach at issue in the article, preparing the way for the hybrid model developed over the two approaches. In brief, the articles cited have helped lead to the present top-down (construct)/bottom-up (symptom network) causal model relating symptom and mental disorder. In addition, the review provides comments that prepare elaboration of the present model.

## Literature Review

The literature review concentrates on the disorders of posttraumatic stress and depression, in particular. It especially analyzes the research by McNally et al. (2) for the former, and Wigman et al. (14) for the latter.

### Posttraumatic Stress Disorder

#### Dimensions

In a literature review and conceptual analysis, Rosen and Lilienfeld (30) evaluated the core assumptions of PTSD. They found that research findings provided no compelling or consistent support for its core assumptions. They queried whether it is a diagnostic category that should be kept in the DSM. In a later publication, Rosen and colleagues called for a process of active questioning to determine its validity (31). That being said, the literature has consistently engaged in scientific investigation of PTSD and its validity. Previously, I noted that the DSM-5 field trials found it to be reliable. In the following, I examine one aspect of its validity – concerning its symptom structure. The review will show that, rather than the current four-cluster model for PTSD in the DSM-5, models with more factors better fit the 20-symptom symptom list for PTSD as found in the DSM-5. In particular, a seven-factor model has been found to be the most powerful and, moreover, it has been found to have associations indicative of its clinical and theoretical value (32, 33).

Table 1 indicates the basic symptoms of PTSD both in the psychiatric diagnostic (nosological) manual, the DSM-IV [Diagnostic and Statistical Manual of Mental Disorders, Fourth Edition; (34)] and the DSM-5, and how the symptoms of PTSD are organized into clusters in these manuals. Moreover, the factor analytic research on how the symptoms cluster have not supported the way the DSMs have parsed the PTSD symptoms into clusters. In this regard, as mentioned, the most recent research of PTSD symptom clustering has indicated that a seven-factor model fits best how the 20 symptoms of PTSD in the DSM-5 organize into clusters [Ref. (32); and replicated by Wang et al. (35); as summarized in Ref. (36)]. Furthermore, the research supports a dissociative PTSD subtype. In this regard, I have argued that there are really eight dimensions to consider in PTSD as described in the DSM-5 (36). Finally, the tables show my approach to which are core symptoms rather than non-core ones among the PTSD symptoms in each of the eight clusters involved in PTSD and the dissociative subtype in the DSM-5 (36).

**Table 1:** **DSM-5 PTSD symptom cluster model (seven) and the dissociative subtype (one), with hypothesized core/non-core symptoms specified**.

Number	Symptom	Cluster	Non-core	Core
**PTSD**				
1	Memories (intrusive)	Re-experiencing	–	✓
2	Nightmares (recurrent)	Re-experiencing	✓	–
3	Dissociative reactions/flashbacks	Re-experiencing	✓	–
4	Emotional reactivity (heightened; to signals)	Re-experiencing	✓	–
5	Physiological reactivity to reminders (marked)	Re-experiencing	✓	–
6	Avoid thoughts/feelings/memories (reminders)	Avoidance	✓	–
7	Avoid external reminders	Avoidance	–	✓
8	Amnesia: inability to recall important aspects	Negative affect	✓	–
9	Negative beliefs (persistent, heightened)	Negative affect	✓	–
10	Self/other blame (persistent)	Negative affect	✓	–
11	Negative emotional state (persistent)	Negative affect	–	✓
12	Loss of interest (marked)	Anhedonia	✓	–
13	Detachment	Anhedonia	–	✓
14	Restricted positive affect	Anhedonia	✓	–
15	Irritability/anger	Externalizing behavior	–	✓
16	Reckless/self-destructive	Externalizing behavior	✓	–
17	Hypervigilance	Alterations in arousal and reactivity	✓	–
18	Startle (exaggerated)	Alterations in arousal and reactivity	–	✓
19	Difficulty concentrating	Dysphoric arousal	✓	–
20	Sleep disturbance	Dysphoric arousal	–	✓
**Dissociative subtype**				
1	Depersonalization	Dissociation	–	✓
2	Derealization	Dissociation	✓	–

*The table indicates the 20 symptoms in the DSM-5 [Diagnostic and Statistical Manual of Mental Disorders, Fifth Edition; (1)], and the 17 in the DSM-IV [Diagnostic and Statistical Manual of Mental Disorders, Fourth Edition; (34)]. There have been changes in wording for some of the symptoms from one version to the next, and the abbreviated versions in the table refer to the DSM-5 symptom list. One of the symptoms in the table (sense of foreshortened future) applies only to the DSM-IV. The table also gives the arrangement of the symptoms into clusters in the DSM-5 and in the DSM-IV. There are four clusters for the former and three for the latter. The DSM-5 clusters essentially involve splitting the DSM-IV avoidance/numbing cluster, consistent with the factor analytic model on the DSM-IV factor structure (37). There are other competing models. Moreover, other research (38) points to a separate cluster related to the DSM’s dissociative subtype. The table indicates which of the symptoms for each of the eight clusters in the table appear to be predominant, essential, or core (39)*.

*Adapted from Young (36)*.

The factor analytic research on PTSD uses confirmatory factor analysis (CFA), which is based on *a priori* models that are tested. Until recently, only four-factor models had been supported, but the work of Elhai et al. (40) had shown that there might be five factors involved in PTSD, and more recently two six-factor models were tested and supported (32, 41) before they were combined in the seven-factor model. Therefore, the understanding of PTSD is becoming more refined and each cluster found represents some psychological construct related to it (e.g., re-experiencing, avoidance, hyperarousal/reactivity). This research is valuable for differentiating models of PTSD at the level of the higher-order constructs that comprise it, because working with 17 to 20 symptoms or so is quite difficult clinically. This is one reason why I tried to isolate the core symptoms in each of the clusters involved.

Zelazny and Simms (42) conducted CFA in a study of psychiatric outpatients assessed using DSM-5 symptom criteria of PTSD. For both samples studied (those meeting either criteria in interview or a subthreshold stressor), the best fit of the data involved the above-mentioned seven-factor model. However, a new six-factor model also fit well the data (named alternate dysphoria, in which difficult concentrating and sleep problems are removed from the dysphoric arousal factor in these models and placed in the dysphoria factor).

Clearly, the research continues on the factor structure of PTSD. That being said, the lack of final response to the question cannot be taken to invalidate PTSD.

#### Networks

Partly in reaction to the complexity of working with long lists of symptoms, researchers using the symptom network approach to PTSD are attempting to discern how symptoms coordinate into nodes and their relations, referred to as edges. Also, they seek the centrality of symptoms in networks, such as in measures of betweenness. The approach statistically is quite different than that of CFA, which focuses on underlying constructs. In network approaches, the nodes and edges are the foci, and symptom themselves in their networking create and influence each other outside of any putative underlying construct.

In the network approach, symptoms covary, or couple variably, and affect each other through feedback loops, homeostatic relations, and so on, allowing sensitivity to individual differences in symptom expression and their causality. For example, an episode of PTSD would follow a course related to symptom nodes in the network “turning on” and “transmitting their activation” to nodes connected to them.

McNally et al. (2) presented a network approach to the symptoms of PTSD. They conducted a questionnaire study of survivors of a 2008 Chinese earthquake, with over 360 respondents. They used a translated version of the PCL [Posttraumatic Checklist – Civilian; (43); Mandarin Chinese version; (44)]. The questionnaire is keyed to the DSM-IV. According to the questionnaire, 38% met the criteria for probable PTSD (5 years after the earthquake when the data were gathered).

The data showed that with exclusion of results at *r* ≤ 0.30, strong associations become more evident, for example, for hypervigilance and startle and also avoidance of thoughts and activities (about the trauma and associated with it, respectively). Numbing and dissociation symptoms were strongly linked (loss of interest in enjoyable activities; feeling distance from others, respectively). Finally, nightmares, flashbacks, and intrusive memories related to the trauma were tightly linked. The authors noted that these various symptom linkages appear related to the three DSM-IV symptom clusters of hyperarousal, avoidance/numbing, and re-experiencing, respectively. However, other symptom linkages did not conform to these DSM clusters – those of startle-concentration problems, and anger-concentration problems.

Other results included that concentration networking indicated that two re-experiencing symptoms were not connected to the others (physiological reactivity, feeling upset at reminders), but quite connected to each other. Centrality calculations showed that a highly central symptom concerns perceiving the future as foreshortened. Overall, the authors concluded that hypervigilance, future foreshortening, and sleep appear predominant symptoms in PTSD symptom network analysis, with multiple symptom linkages involved, including some not previously considered.

To conclude this section of the paper, I note that in Young et al. (45), I attempted to show how a network model of PTSD symptoms could distinguish primary (core), secondary, and tertiary ones. That work indicates that network thinking can be applied to mental disorder in multiple ways.

### Depression and Other Disorders

Bielczyk et al. (15) adopted a similar model for major depressive disorder. According to them, causal relations in network dynamics are the cause of clinical constructs such as depression.

Bielczyk et al. (15) added a role in depression of attractor dynamics and also for the regulation of excitation–inhibition balance across brain circuits. These latter concepts are quite consistent with my own (6), in that I argue that NLDST can help explain shifts to health and illness attractors and that activation/inhibition coordination is an important mechanism at all levels in brain–behavior relations.

Conway and Kovacs (46) have shown how the field of human intelligence is moving away from the traditional latent psychological construct model (*g*, general factor of intelligence) in which *g* is considered a causal general ability, to new models that interpret *g* as an emergent property reflecting the positive correlations found among test scores. This research shows how the concept that underlying constructs need to be complemented if not replaced by other models is gaining traction in areas other than psychopathology.

These newer models are “formative” ones, and not the traditional “reflective,” essentialist, or “entity realism” ones. In formative models, there still are psychological constructs, but as causal effects or consequences rather than causal initiators.

Conway and Kovacs (46) concluded that hybrid models of intelligence exist, and they are partly reflective in nature and partly formative, too, such as found in their own “process overlap” theory. As has been emphasized, for the topic of psychopathology, the present work also is proposing a hybrid reflective (top-down) and formative (bottom-up) causal model of the relationship between symptom and illness. The model that I have created derives from the seminal work of McNally et al. (2) and also that of Wigman et al. (14), presented next.

Wigman et al. (14) examined data gathered by experience sampling methodology (ESM) in a pooled sample (*N* = 599) of three groups (depression in past; current status mild; psychotic symptoms, with disorder diagnosed; controls). Participants were given wristwatches that beeped quasi-randomly 10 times per day over a period of 5 to 6 days (depending on the particular sample). The signal required them to fill in a self-assessment diary. The focus of their study was to analyze the relations of participants’ responses, as given on a 7-point Likert scale, for five items, which were – at this moment, I feel: cheerful; content; insecure; down; suspicious.

Wigman et al. (14) reviewed the top-down psychological construct approach to mental disorder. As shown, in this approach, mental symptoms are viewed as being caused by underlying constructs. In contrast, the bottom-up approach that they reviewed maintains that psychopathology involves a complex interacting network of components. At the symptom level, this approach views mental states as nodes that, when activated, might trigger other mental states (47). Symptom networks might be non-linear in their mutual effects, reciprocal, with feedback loops, vicious circles, and increased connectivity.

Despite pointing out the major differences in the two models of how symptoms and disorder might relate, Wigman et al. (14) did not contrast in a direct fashion one model vs. the other. Rather, they compared mental state network structure over groups having the different diagnoses mentioned to healthy controls. Also, they sought clusters of network components across network data. Note that the network characteristics analyzed involved centrality indices: node strength, outward degree, inward degree, closeness, and betweenness.

Perhaps because the first type of analysis undertaken involved group comparison and the second transdiagnostic analysis, the authors referred to the cross-group network analysis as top-down even though it makes more sense to refer to network analysis as bottom-up and they referred to the principle component analysis as bottom-up even though it typically would be referred to as top-down compared to network analysis. In short, I query whether their approach by Wigman et al. (14) allows for the hybrid reflective–formative conceptualization of mental disorder and their relations to symptoms. It seems that all they did was analyze the data involved with the two types of statistics typically associated with one approach or the other, but not in the way that the statistics are typically used in this type of research. Careful analysis of their results in what follows confirms this impression.

The results in Wigman et al. (14) showed that having a diagnosis led to more strongly connected moment-to-moment mental state network structures, and more so for depression relative to psychosis. For example, in depressed patients, there were many more interconnections between negative and positive emotions, unlike the case for the group with psychosis, for which connections like this were rare. In the latter group, there appears to be two separate loops of mental state, one negative and the other positive. In terms of the connectedness measurement, it was higher in the group with depression, e.g., in terms of node strength and inward and outward degree. Depressed individuals had the highest node strength. Finally, the comparison group had the least connections going to or coming from negative mental states.

As for the principal component analysis results, seven high-order components emerged. They were based on loadings over associations such as mental state at time *t* − 1 and what follows at time *t*. For the first factor, all the loaded associations began with a positive emotional state at time *t* − 1. Therefore, the authors interpreted it “impact of positive mental state.” The second factor seemed to reflect the negative impact of feeling down on other mental states, and so on. A primary result was that compared to the controls, the two psychiatrically disordered groups obtained higher scores on the component of “impact of insecure,” suggesting that this component might be a general one in mental illness in multiple dimensions of psychopathology. The authors noted that the network paradigm appears to be a useful one in mapping transdiagnostic processes in mental state.

Wigman et al. (14) concluded that individuals with the same diagnosis might exhibit substantially different symptom patterns. Moreover, the concept itself of separate diagnoses might be problematic in that psychopathology might reflect one underlying explanatory principle – that of mental state interconnectivity underlying symptoms. These conclusions are quite accurate, but they might reflect the manner in which the analyses were conducted rather than anything like a genuinely hybrid model of reflective and formative models. In such an approach, psychological construct and network analyses would be conceptualized as equal and interacting reciprocal causality mechanisms of the relationship of symptom and psychological construct, and not be considered hybrid simply because a principal component analysis was applied to network data. In the following, I attempt to create such an integrative model of symptom–mental disorder relations. In the latter approach, only the data analysis methods are hybrid, not the conceptualization.

This penultimate section of the article follows next and outlines a genuine hybrid model of symptom–mental disorder relations from a bottom-up–top-down perspective. Once the model building is complete, the article considers further the nature of symptoms, for example, in terms of the value of perceiving them as individualized mental content and meaning.

## The Hybrid Symptom–Mental Disorder Model

Specifically for this section of the article, I re-introduce the bottom-up and top-down models of the symptom–mental disorder relationship. Then, I show how the two models can reciprocally interrelate.

### Modeling

In models of symptoms and mental disorder relations, one set of models concerns higher-order (latent variable) constructs (e.g., PTSD) that cause or influence in a top-down manner the lower-order manifest symptoms and their clusters (which in turn might be an intermediate level of influence on symptoms). In contrast, according to network models, cluster/symptom interactions cause their pattern of expressions and the term associated with mental disorder (e.g., PTSD) is a representation of the symptoms and their interactions rather than being a causal influence on their manifestation.

**Figure 1:**
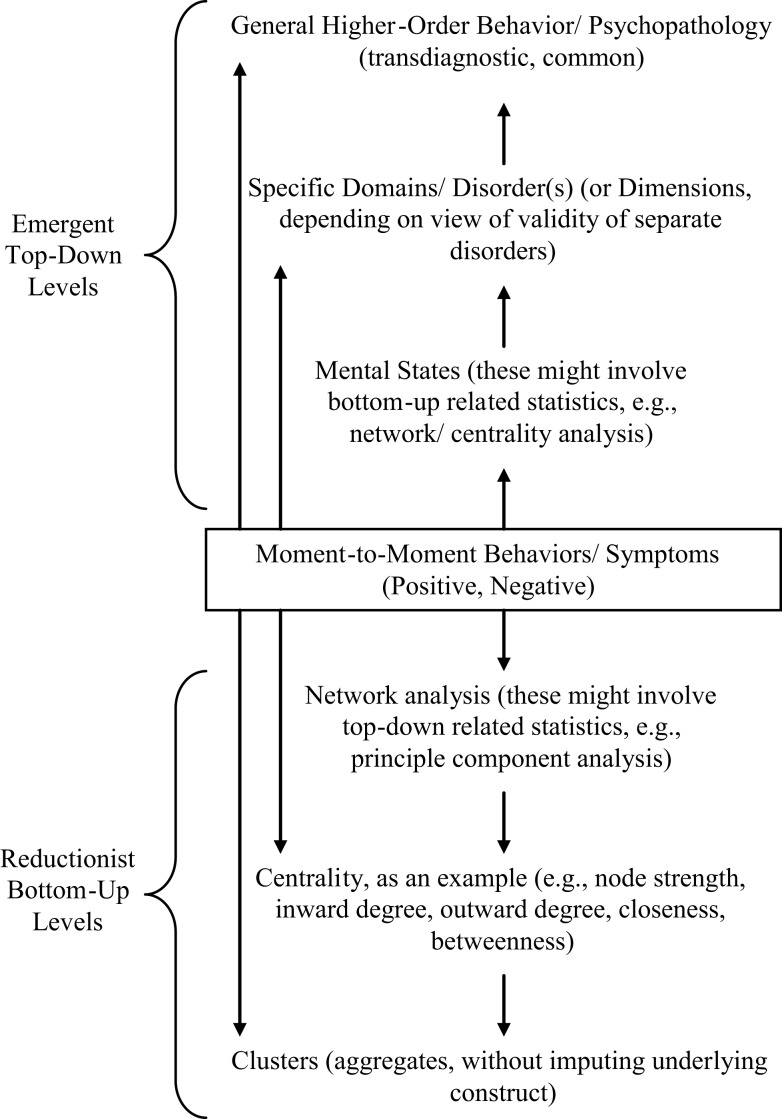
**Top-down and bottom-up levels in behavior/symptom normality/psychopathology**. The figure illustrates the complementarity of top-down and bottom-up models of the causal relationship between symptoms and mental disorder. In systems, different levels might exist with higher-order levels emergent or distinct in characteristics from lower-order ones on which they are built. In terms of the constituents of the levels, they might be single elements, patterns, or networks, or even superordinate structures. Statistical techniques, such as network analysis or principle component analysis, can be applied to both emergent, higher-order levels and more basic ones. In terms of psychological constructs, at the higher-order emergent levels, in psychopathology, they might be specific disorders, dimensions, or overarching constructs (e.g., internalizing/externalizing disorders).

One way of accommodating the different views on psychopathology of what constitutes bottom-up and top-down processes is to consider a systems model with different levels (see Figure 1). Systems thinking is best exemplified by NLDST [e.g., Ref. (3, 16)], in which different levels of a system might interact and even be created in the interaction, just as different elements in any one level of the system (or of the system as a whole) might interact and even create new elements. Moreover, changes in system state might take place because of minor perturbations when the system is at far-from-equilibrium, while living systems generally might be poised at this state preparatory to change because of its adaptive value.

Figure 1 illustrates the difference between a hybrid conceptualization of symptom–mental state relations and a hybrid statistical analysis of the relations. There is no reason why it cannot be the case that with each of the classic top-down, psychological construct approach and the bottom-up, symptom-driven approach, there are both network and cluster statistics that could be used.

Although powerful, the network approach to symptom causality and connection is more descriptive than mechanistic. It might indicate that symptoms connect and even in unique ways compared to conjectures and findings based on other approaches. However, the causes of the connectivities involved are not specified except by indicating that the symptom interactions are the cause. This might represent tautology, although I am sympathetic to the lower-order, grounded, and micromoment dynamics producing the connectivities. We need to know which symptoms emerge as predominant in any one moment of time, and the work of Wigman et al. (14) provides methods for tackling this issue. Moreover, they also refer to dynamic temporal processes in network expression.

Nevertheless, at another level, systems theory could tell us more about emerging connectivities over symptoms and their relations to mental state. In this regard, the work of Bielczyk et al. (15) indicates that mechanisms that might cohere symptoms (or repel them) might act through dynamical system processes [including activation/inhibition balancing, which is a concept central in my work: (3, 6)]. Symptom system dynamics can be measured in different ways in dynamical systems approaches compared to network ones, for example, in terms of control and order parameters and of exponents related to bifurcation points in which systems split into new attractor regimes or chaotic–antichaotic adaptive systems, fractal patterns, and so on.

That being said, the micromoment approach to symptom connectivity at times *t* − 1, *t*, *t* + 1, etc., could inform these analyses in complementary ways. For example, patients might have a more powerful symptom at any one time among their suite of symptoms, or one symptom might lead the way at any one moment in bringing a subthreshold one to disorder (and perhaps disability). As yet, there is no clear integrative model of how any one symptom might become primary in these senses at any one moment, although, as shown, the work of Wichers et al. (27) has made strides in these regards.

The symptom complex of the patient is crucial, as are symptom linkages over individualized patterns, or the network of nodes/edges (relations) expressed by the patient over time. Based on this approach, the clinician might develop individual mappings of the dynamic evolution of symptoms over sessions and apply individualized approaches to intervention and treatment.

To conclude this portion of the article, hybrid conceptualizations to date on the relationship of symptoms and disorder have much to offer, but there might be conceptual limitations in the work that bar further progress. In this regard, current hybrid models [e.g., Ref. (14)], as argued above, might not allow for genuine reciprocity between the causal effects of the higher-order construct and the lower-order symptoms. Only by avoiding to equate any statistical modeling with conceptual ones and also by finding a common conceptual umbrella for both types of models can a genuine hybrid one over them be constructed. The next section presents a systems model-informed hybrid model of symptom–mental disorder relations based on these premises.

### The Model

**Figure 2:**
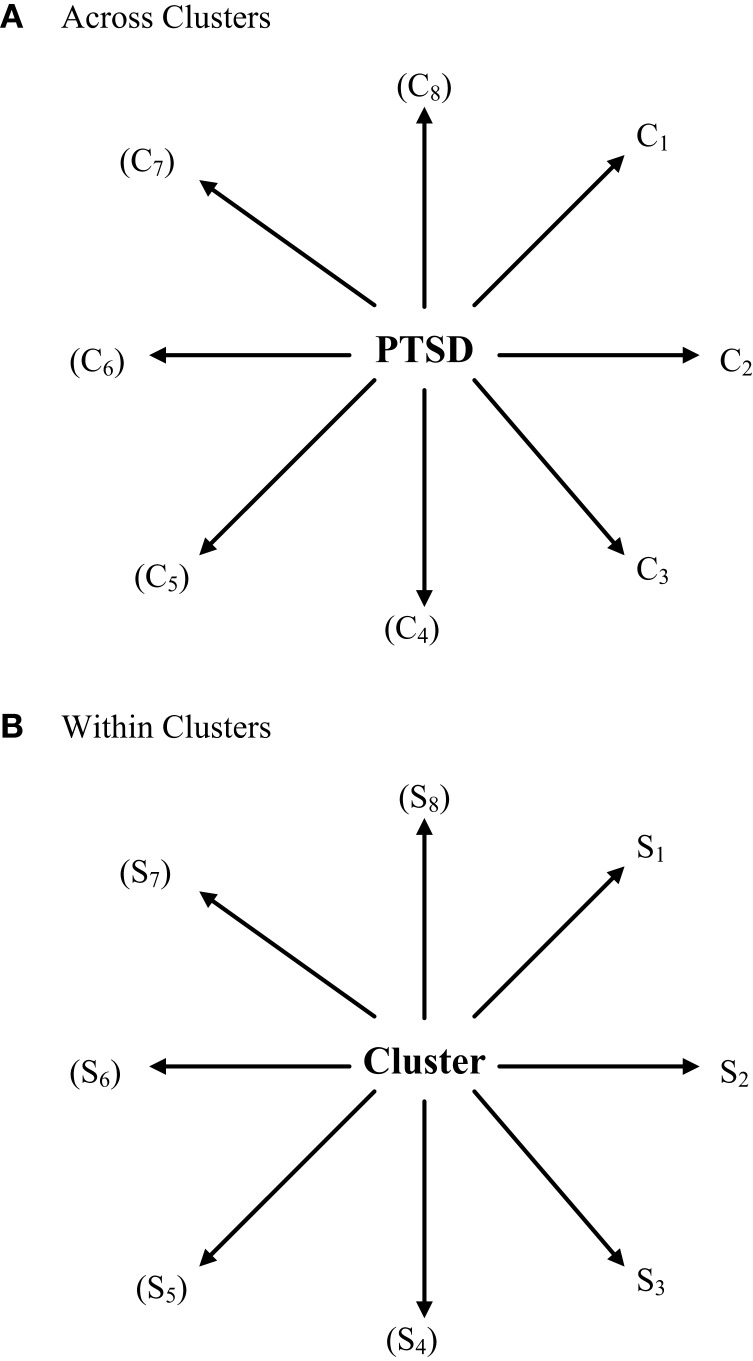
**A latent variable construct (top-down) causal model of PTSD symptoms (S) and clusters (C)**. **(A)** Across clusters, **(B)** within clusters. In latent variable or construct models of psychological phenomena, an “essential” underlying psychological entity, trait, characteristic, or superordinate attribute is considered as a valid higher-order behavioral reality that is not caused by or conditioned by the lower-order behaviors/symptoms associated with it but, to the contrary, conditions or causes in a top-down manner how they are manifested (in context, over time/development). Mental disorders might have several clusters and each can be characterized as a quasi-dependent sub-disorder that conditions/causes its associated symptoms. In this model, individual differences derive from the overarching construct involved and not from the manifested symptoms themselves, which merely reflect, in their patterns, the higher-order individual differences involved.

**Figure 3:**
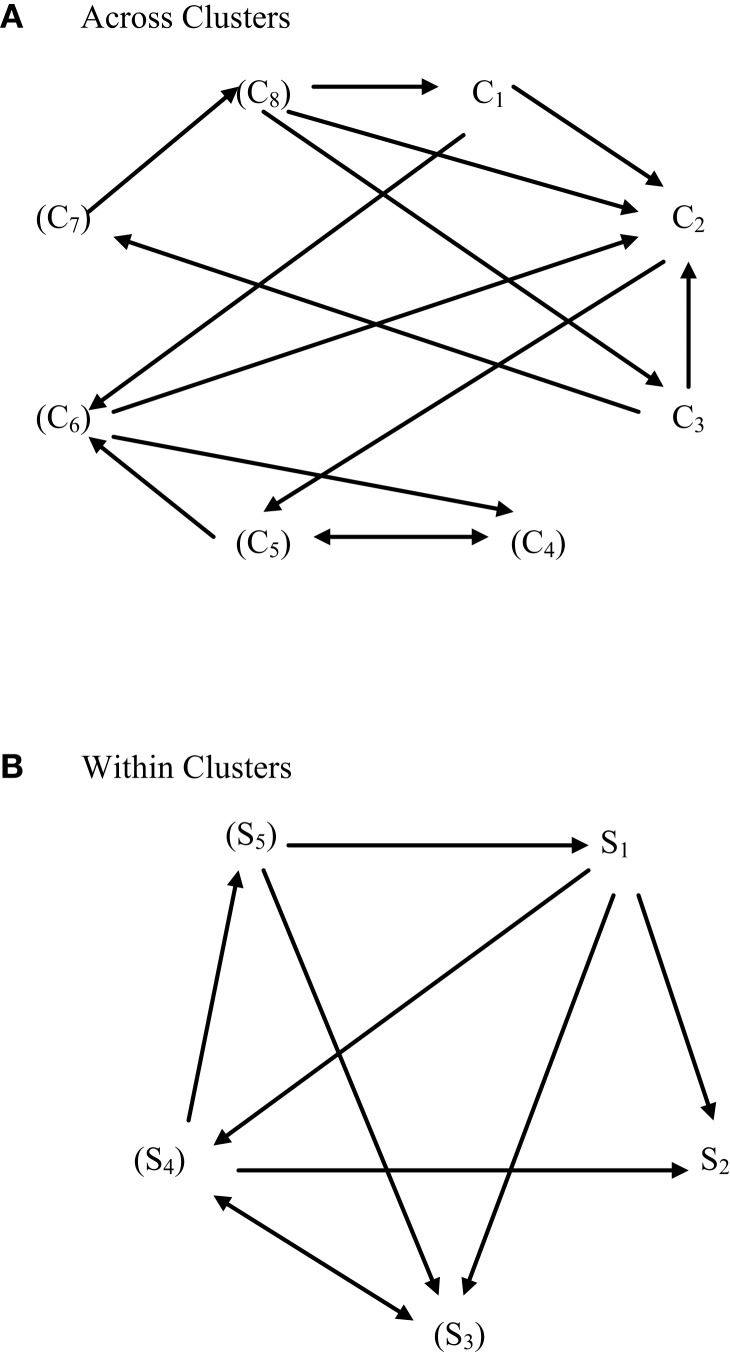
**A symptom-interactive (bottom-up) causal model of PTSD symptoms (S) and clusters (C)**. **(A)** Across clusters, **(B)** within clusters. In “non-essentialist” system-interactive or behavior/symptom network, connective models, behaviors/symptoms interact amongst themselves and constitute the cause of the pattern of behaviors/symptoms expressed. For example, if sleep is poor, other symptoms might be exacerbated. Individual differences in behavior/symptom expression derive from the behavior/symptom interactions in context (and over time/development). There is no higher-order “essential” (latent) psychological variable, construct, entity, trait, characteristic, or attribute that influences the behavior/symptom interactions. If terms relating to these levels of behavior are used in this model, it is only to represent the interactions and not as a factor that causes or influences them. In this regard, behaviors/symptoms in interaction do so at a level that is bottom-up rather than top-down.

Figures 2 and 3 present core material of the present model of how symptoms and mental disorder interrelate in a hybrid fashion. The second of these two figures specifies how the concept of emergent circular causality (3) can be applied equally to the bottom-up and top-down approaches to causality. Specifically, Figures 2 and 3 depict the difference between the latent variable/psychological construct model of the relationship between PTSD and its clusters/symptoms and the symptom-interactive or network model.

In considering development of a genuine hybrid model over the construct and symptom network approaches to how symptoms and mental disorder relates such that construct and symptoms causally interact, primacy should not be given to either component. Moreover, the statistical models that one might choose to work within each paradigm constrain the model building involved.

The next section of the article specifically demonstrates how a more integrative model of the reflective construct and formative network models could be constructed for the question of ­symptom–mental disorder relationship. It avoids some of the pitfalls of prior attempts to do the same. Nevertheless, it is an initial conceptualization that itself has limitations, such as not yet being mathematically grounded nor empirically tested.

**Figure 4:**
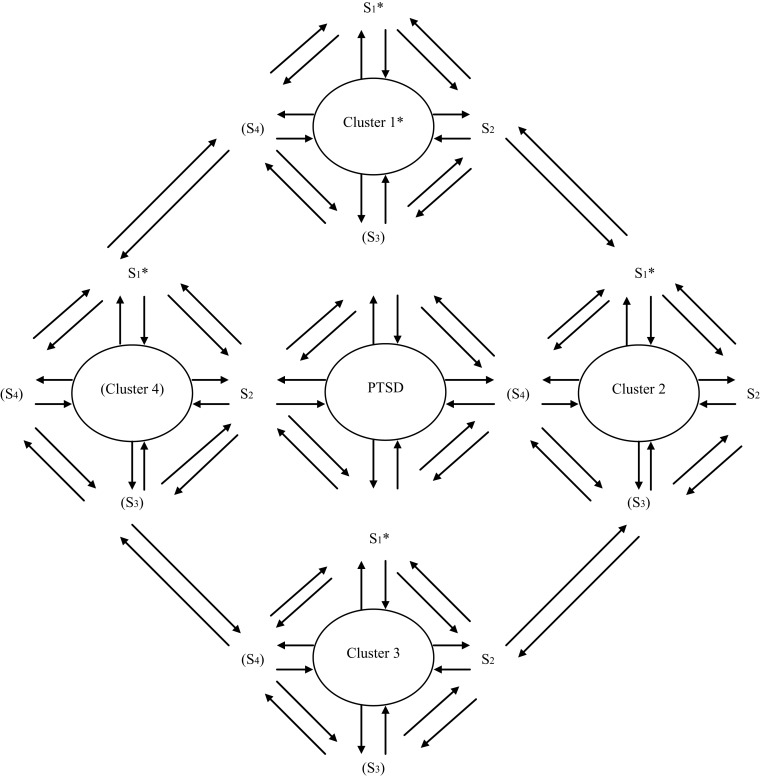
**Integrative causal symptom-construct model in mental disorder**. The figure depicts the relationship between symptoms and mental disorder (or a symptom cluster of one) as dynamically reciprocal in causation. The mental disorder constitutes an underlying, higher-order level in the patient’s mental state symptoms, while the symptoms interact at lower levels of the system, with both the top-down and bottom-up influences dynamically influencing each other in context and over time. Note: the parentheses indicate that PTSD might have only three clusters (as in the DSM-IV), and a cluster might have only two symptoms. Of course, depending on the disorder involved either might have more items (i.e., clusters or symptoms, respectively). Of the clusters in any mental disorder, for their symptoms, it would be beneficial to specify which ones are core/primary. For the model presented in the figure, these could be the first clusters or symptoms that are specified by the asterisks.

**Figure 5:**
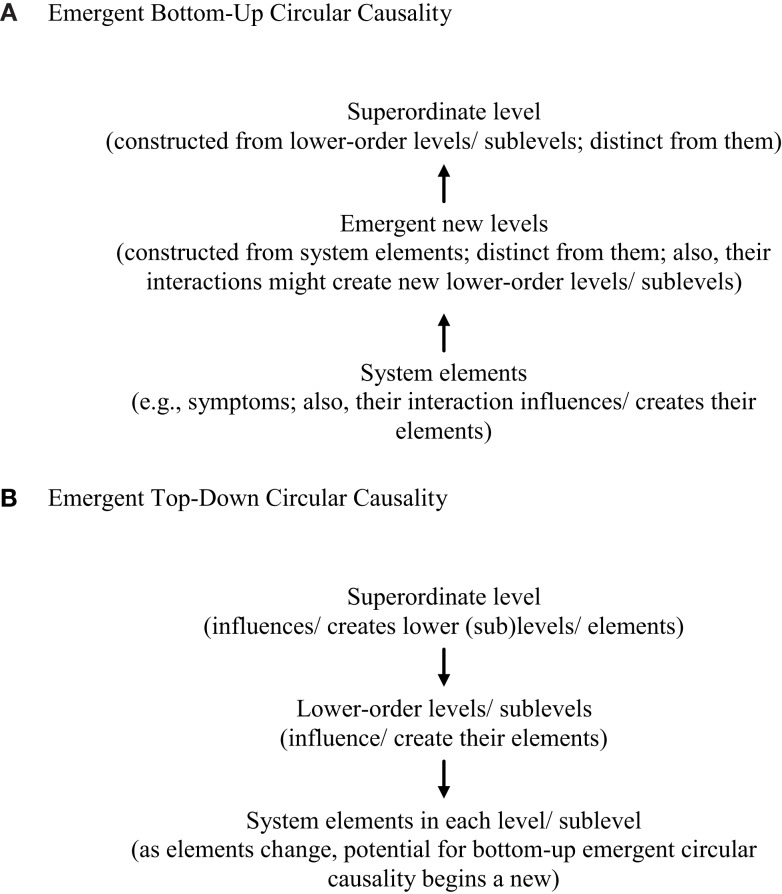
**The interaction of top-down and bottom-up emergent circular causality**. **(A)** Emergent bottom-up circular causality, **(B)** emergent top-down circular causality. Note: (1) Configuration/pattern changes possible, too, within and between (sub)levels. (2) Bottom-up and top-down causal processes work together reciprocally in system causality. The figure illustrates the dynamic interaction of bottom-up and top-down processes both within and across levels in a system, including the possibility of emergence of new symptoms, levels, and sublevels. It also indicates the change of patterning or configuration possible within and between levels in the system dynamics involved. Briefly, as system elements (e.g., symptoms) or levels/sublevels interact, they might influence/create their configuration/patterning, expression, or even *de novo* emergence. This process may occur both through movement from lower to higher levels in the level hierarchy involved (bottom-up), or from higher to lower levels (top-down), or reciprocally in both ways. In essence, the figure clarifies that, in system function, bottom-up processes work both within and between levels, as do top-down processes.

Figures 4 and 5 present a genuine hybrid reflective and formative model of causality over mental symptom and disorder. For any one construct or cluster, there is not only influence/creation downward to symptoms but also feedback upward from symptom interactions to construct/cluster. Moreover, these top-down and bottom-up models function at multiple intermediary levels (intermediate, superordinate) and the interactions can take place not only horizontally (among symptoms; among levels/sublevels; and their configurations/patterns) but also vertically (downward or upward over (sub) levels).

Therefore, causality does not reside in one nexus node, level, element, element (sub)set, construct, or multiple aspects of these constituents of the symptom and disorder but in all the rich dynamical systemic interactions and reciprocal influences among them. Symptoms have causal effects on each other but constructs have causal effects on them. Constructs, such as mental disorder, are not ephemeral, reducible entities to symptoms, but emergent, irreducible entities that can affect and even initiate the symptoms. They reflect dynamical system characteristics, and can take on a life of their own at higher-order levels of a system. Perhaps they are not directly observable, but their role can be inferred and the mechanisms that being them about are increasingly understood.

In short, emergence is a common construct in systems theory, but in my approach to it, circular causality constitutes an important driving mechanism in emergence (48). That is, as system levels interact with one another, new ones can emerge at higher orders, and they can become overarching and overriding drivers of behavior and symptom expression (3, 6). Specifically, I had written in Young (3) that in “circular emergence” different levels of systems can form and integrate, with higher-order ones gaining degrees of freedom through their flexibility even as their degrees of freedom are constrained through the intercoordinations involved. Also, I noted that activation/inhibition coordination can serve as the critical mechanism in stabilizing systems, in keeping them at the cusp of change, and in recreating equilibrium after they change.

## Conclusion

First, the article has provided background information, such as relevant definitions and issues related to nosology, causality, and network and construct models of symptom–disorder relations. Then, it reviewed the relevant literature in the field, tackling alternative models and trying to disambiguate them. Next, it gave a genuinely hybrid model for the relationship of symptom network and psychological constructs in mental disorder.

To conclude the article, in the next section, I return to considering the nature of symptoms by querying their unconscious, subjective, descriptive, and meaning side compared to their conscious, objective, and reductionist universal causal side. I present a novel model that addresses the question in an integrated manner.

One could ask even whether overarching illness entities could impact symptoms, and that mental disorders could be reducible to symptoms sets, as in the DSM-5. One answer to this conundrum would be to abandon the DSM-5 because of its multiple critics [see Ref. (7, 49)]. For them, the DSM-5 has theoretical, epistemological, and social weaknesses; was the result of a chaotic revision process; does not consider sufficiently the causality related to the listed mental disorders; they are artificial; and so on. However, continued research and revision of its categories could be improving its clinical usage.

Psychiatry needs to address critical questions on the nature of symptoms and mental disorder but, at the same time, balance scienticism and skepticism, or create hybrid models that integrate them and go beyond them. We need pause for thought in evaluating the relative roles of hermeneutic insight and causal explanation in psychiatry (Verstehen, Erklären, respectively). Whether we accept that symptom meaning/content, or phenomenology can be influenced by hermeneutic insight, “Verstehen” has important consequences. Are symptoms and their meaning/content only what can be observed, and therefore, reduced to what is measurable, or are there other levels to consider? In this regard, symptom meaning or content might be a higher-order level in the symptom/disorder complex, whether the symptoms are observed or self-reported. Moreover, observed and self-reported symptoms could be tapping different patient realities, and what might these differences mean for symptom meaning/content? For cause, are reductionist, biological views used to explain symptoms/disorders rather than higher-order mental content or constructs? Can the latter causally influence lower-order (and more easily observed/self-reported) symptoms (Erklären)?

**Figure 6:**
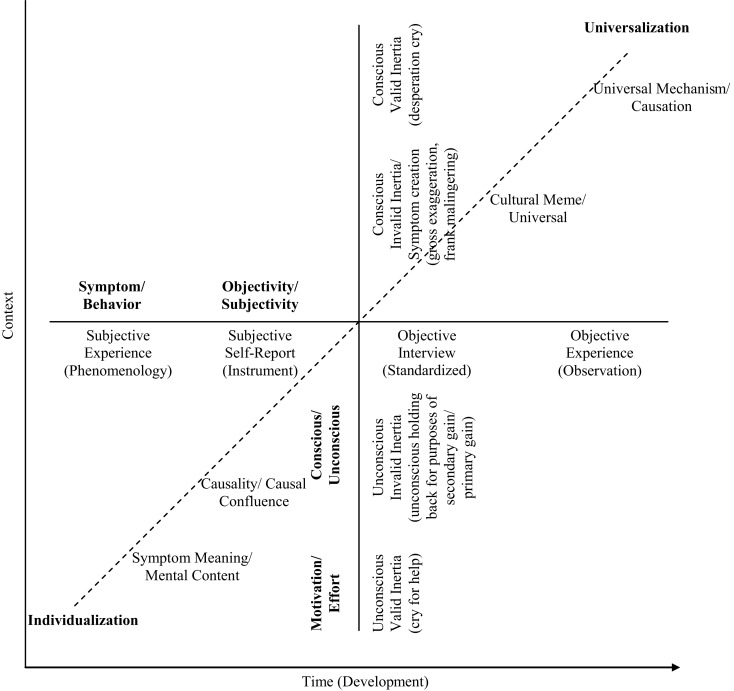
**The dimensions of symptom expression in psychiatry**. The figure places symptoms along three dimensions that concern: motivation/effort (are symptoms valid?); subjectivity/objectivity (e.g., phenomenological, empirical); and individuality/universality. (a) Symptoms might not be valid, or they could be ones that should not be taken at face value. For example, about the former, there might be conscious malingering for secondary gain that is taking place. About the latter, there might be unconscious influences at work; for example, an unconscious cry for help could take place or a conscious desperation cry. (b) Also, symptoms vary in terms of they are ascertained subjectively or objectively. For example, they might derive from the person’s self-report, in particular, or from the evaluator’s more controlled efforts to observe/discern them. (c) Finally, symptoms vary in their individuality/universality. At one extreme, each symptom has a personal meaning or value for the patient/client that is unique to her/his history/narrative/experience. Or the causes of the symptoms constitute a unique constellation. On the other hand, they might indicate some cultural universal or meme/mythic narrative. In addition, they might reflect a universal causal or mechanism in behavior to which we are all subject and in constant, invariant ways.

Figure 6 presents a model of symptom expressions that illustrates the difficulty in addressing these types of questions, while proposing a nuanced solution. On the one hand, symptom meaning and causality do not necessarily stand in opposition. For example, at the level of individualization and universalization, symptoms could be unique to the person’s history and current mental content, as well as unique in the coalition of forces that had created them. As well, symptoms could reflect universal themes and concerns, and also reflect standard common causal mechanisms.

Ultimately, both individual and universal mental content and symptoms might not be what they appear, either to the person expressing them phenomenologically and subjectively or to the observer using empirical methods, e.g., in observations, interviews, self-report questionnaires, in discerning them. Both subjective and objective understanding of symptoms might approach their reality.

Certainly, a complicating factor in all these regards relates to the play of unconscious processes in symptom creation and expression. This could apply in the sense of (a) classic Freudian repression, (b) automaticity in thought without deliberative reflection or insight, or (c) a lack of awareness of the overall system in which the symptoms are embedded.

Specifically for the area of PTSD and some other related conditions/disorders that are subject to legal dispute (e.g., chronic pain/somatic symptom disorder), the answers to these types of questions are complicated by court considerations. One needs to veer toward the more objective side as much as possible in order to vet possible confounds, such as malingering and unconscious influences on clinical presentation and self-report. Figure 6 illustrates that intention is very difficult to evaluate and can never be evaluated uniquely by test results or clinical interview. Young (13, 36, 39, 50, 51) has presented work relevant to the question, calling for a scientifically-informed comprehensive impartial approach to assessment in these types of cases.

Ultimately, the network approach to symptom and mental disorder relationship addresses some of the issues raised about individual insight vs. universal explanation in symptomatology because, in this view, how symptoms interact becomes the seat of causal explanation and understanding. Nevertheless, in my hybrid model, one needs to consider top-down psychological construct influences on symptoms as much as their bottom-up interactions, so that their meaning and causation lay in not only symptom networking processes but also in higher-order levels in the symptom structure and the causes associated with them.

Often issues in our field are presented as a dichotomy, or in black and white. For example, for the causes of behavior, too often they are phrased as Nature vs. Nurture. Yet, behavioral causation reflects an interaction of biological, personal (e.g., self, free will belief), and environmental factors (6). Similarly, for the issue of scientism vs. skepticism and how it relates to considering symptoms in terms of individualized meanings or universal (read reductionist) causal mechanisms (Verstehen, Erklären, respectively), the opposition is presented too simply. The question of whether the nature of symptoms are either more unconscious, subjective/phenomenological, and meaningful in content or more conscious, observable, objective, and expressions of universal causality might be one that masks a greater underlying complexity in understanding them, such as in the ­three-dimensional model presented in the figure.

In the article, I have presented an integrated top-down (psychological construct)/bottom-up (symptom network interaction) model of the relationship between symptom and disorder. The same model can be applied to understanding the relationship between mental content and their causes. Because of the multiple levels in systems of behavior, emergent contents can develop at higher-order levels that are not totally reflective of, reducible to, or transcribable from the lower levels, including of the causes involved. Mental contents, such as beliefs, emotions, and desires, can emerge and influence symptoms that might be closer to the lower-order biological or physical substrate, including neurobehaviorally, because of the process of circular emergence and the creation of higher-order levels in behavioral systems that the process allows.

In this regard, one example of higher-order mental state influences on lower-order symptoms is found in the how catastrophic and hopeless thought and related cognitive and emotional processes could cause downward spirals in helplessness and amotivation, and then in the specific symptoms of disorders, such as depression or PTSD. In another example, the personally-exaggerated appraisal of stress that then leads to stress-induced headaches is all too real for many of our patients.

Symptoms and mental disorder co-exist in a system in which all relevant levels need to be recognized and researched. There should be no room for exclusive reductionist or constructionist approaches in understanding them, as both are needed. Reductionism and the search for cause in the most basic biological processes should not be equated with scientism. Nor should seeking emergent phenomena that could influence behavior be treated with skepticism. Behavioral, symptom, and mental content states exist coactively with their causes, and science should examine the relations among all these levels with the clarity that patients deserve.

The present article has presented, hopefully, refined thinking in the area of mental disorder. Further effort along these lines might examine a possible systems model of the definition of mental disorder, one that includes levels for – symptoms, clusters, higher-order mental content, mental disorder, and related concepts such as disability. Similarly, treatment can be conceived systemically, e.g., in terms of cascades that might result from effective treatment shifting the patient into the region of health attractors [e.g., Ref. (27)]. Network concepts can be embedded in systems models and, therefore, the two types of models conceptualized together, and even hybridly, can provide a powerful language for grasping the nature of symptoms, mental disorder, causality, and cure (or treatment).

## Conflict of Interest Statement

The author declares that the research was conducted in the absence of any commercial or financial relationships that could be construed as a potential conflict of interest.
